# Dry habitats sustain high CO_2_ emissions from temporary ponds across seasons

**DOI:** 10.1038/s41598-018-20969-y

**Published:** 2018-02-14

**Authors:** Biel Obrador, Daniel von Schiller, Rafael Marcé, Lluís Gómez-Gener, Matthias Koschorreck, Carles Borrego, Núria Catalán

**Affiliations:** 1 Department of Evolutionary Biology, Ecology and Environmental Sciences, University of Barcelona, Av. Diagonal 643, 08028 Barcelona, Spain; 2 Catalan Institute for Water Research (ICRA), Scientific and Technological Park of the University of Girona, Emili Grahit 101, 17003 Girona, Spain; 3 Department of Plant Biology and Ecology, Faculty of Science and Technology, University of the Basque Country, Apdo. 644, 48080 Bilbao, Spain; 4 Department of Ecology and Environmental Science, Umeå University, Linnaeus väg 6, 90187 Umeå, Sweden; 5 Department Lake Research, Helmholtz Centre for Environmental Research – UFZ, Brückstrasse 3a, 39114 Magdeburg, Germany; 6 Group of Molecular Microbial Ecology, Institute of Aquatic Ecology, University of Girona, Campus de Montilivi, 17071 Girona, Spain; 7 Limnology, Department of Ecology and Genetics, Evolutionary Biology Centre, Uppsala University, Norbyvägen 18D, 75236 Uppsala, Sweden

## Abstract

Despite the increasing understanding of the magnitude and drivers of carbon gas emissions from inland waters, the relevance of water fluctuation and associated drying on their dynamics is rarely addressed. Here, we quantified CO_2_ and CH_4_ fluxes from a set of temporary ponds across seasons. The ponds were in all occasion net CO_2_ emitters irrespective of the presence or absence of water. While the CO_2_ fluxes were in the upper range of emissions for freshwater lentic systems, CH_4_ fluxes were mostly undetectable. Dry habitats substantially contributed to these emissions and were always a source of CO_2_, whereas inundated habitats acted either as a source or a sink of atmospheric CO_2_ along the year. Higher concentrations of coloured and humic organic matter in water and sediment were linked to higher CO_2_ emissions. Composition of the sediment microbial community was related both to dissolved organic matter concentration and composition, but we did not find a direct link with CO_2_ fluxes. The presence of methanogenic archaea in most ponds suggested the potential for episodic CH_4_ production and emission. Our results highlight the need for spatially and temporally inclusive approaches that consider the dry phases and habitats to characterize carbon cycling in temporary systems.

## Introduction

Inland waters are an important component of the global carbon (C) cycle^1^. They intensely bury C in their sediments and emit high amounts of gaseous C species to the atmosphere, mostly as a result of the input and subsequent biological processing of terrestrial organic matter^2^. A myriad of local and regional studies in the last two decades have aimed at quantifying carbon dioxide (CO_2_) and methane (CH_4_) emissions from streams, rivers, lakes and reservoirs, allowing for increasingly accurate global estimates^3–10^. We have gained a better understanding of the processes determining C emissions across wide environmental and geographical gradients^5–9^ but uncertainties regarding the variability and controls of such fluxes in some ecosystems still remain. In particular, C processing in temporary systems that recurrently run dry, a natural hydrological feature of many watersheds in the world, has received little attention^11^. As an example, current global estimates do not consider C emissions from temporary rivers when they are dry^11^.

Small temporary lentic systems are one example of an understudied ecosystem in terms of C biogeochemistry. Small systems in general (i.e. ponds, small lakes and impoundments) are deemed as potential hotspots of C cycling^12,13^ and host high emission rates at different latitudes^10,14–18^. The intense rates of C processing in small systems along with their global abundance^19,20^ (77% of lentic systems have a surface area between 0.002 and 0.01 km^2^) suggest the potentially major role of small lakes and ponds at the global scale^10,13^ and an increasing relevance of their C emissions due to their high sensitivity to warming^21^.

Temporary ponds are small inland water bodies that experience a recurrent dry phase of varying duration, which can imply complete or partial drying^22^. They have unique chemical, biological and physical features and have long been recognised as key elements for biodiversity conservation^23,24^. The few existing studies on C processing in temporary ponds show their relevance as C emitters^17,25,26^. However, their emissions have seldom been measured directly^17^, for CO_2_ concentration has been used to indirectly estimate gas fluxes in most cases. Moreover, the approaches used to date have been restricted to the aquatic phase, so dry habitats within ponds or temporal phases without any water at all have not been considered^10,25,26,31^. In contrast, a recent study on Mediterranean temporary ponds has shown the relevance of CO_2_ emissions from their dry habitats during the flooding phase^17^. These results agree with recent works reporting high CO_2_ emissions from the dry areas of temporary rivers and reservoirs^27–30^.

The paucity of data on C emissions from temporary ponds is paired with a poor understanding of the environmental drivers that shape them. Catalán *et al*.^17^, showed that spatially integrated CO_2_ emissions during the flooding phase of Mediterranean temporary ponds were not related to the properties of the catchment, but rather to the intrinsic properties of the pond such as the amount of organic matter present in water and sediments. In contrast, Holgerson^31^ found that variables related to the processing of terrestrial organic matter largely explained dissolved CO_2_ and CH_4_ dynamics in the water of temporary ponds in temperate forested areas, pinpointing the need for a better understanding of CO_2_ and CH_4_ regulation in ponds.

Here, we quantified CO_2_ and CH_4_ fluxes in Mediterranean temporary ponds along the different phases of their hydrological cycle. We selected ten temporary ponds located on the island of Menorca (Western Mediterranean) covering a wide range of physicochemical, geomorphologic and hydrological properties (Table 1). To characterize contrasting situations along their hydrological cycle, we sampled the ponds in autumn (the *flooding phase)*; in spring when the ponds are mostly full (the *wet phase);* and in summer, when the ponds were entirely dry (the *dry phase*). Moreover, to capture all variability across and within ponds, we sampled the three habitats present in the ponds: *dry* habitats, comprising 1) *emerged-vegetated* sediments (non-inundated areas with presence of vascular plants), and 2) *emerged-bare* sediments (non-inundated areas with bare sediments); and 3) *inundated* habitats (areas with a water layer; *see* Table S1 for habitat areas). We performed direct measurements of gas fluxes in all these habitats separately and integrated them to estimate total fluxes for each pond. We hypothesized that fluxes would be comparable or higher to those in other lentic ecosystems and highest during the dry phase of the hydrological cycle due to large contributions from dry habitats. We also identified the main drivers of the variability in the fluxes by evaluating the roles of a wide set of geomorphological, physicochemical and biological variables such as water and sediment organic matter quantity and composition and microbial community composition in the ponds studied.

**Table 1:** Morphometric descriptors and mean physicochemical characteristics of the studied ponds.

Pond	Max. depth (cm)	Max. flooding area(m^2^)	Catchment area (ha)	Type of hydroperiod and duration of the wet phase on a typical year^46^	TN	TP	Chl-a	Alk	O_2_	EC	pH	Sediment organic matter (LOI; %)	DOC
					(mg L^−1^)	(mg L^−1^)	(µg L^−1^)	(meq L^−1^)	(mg L^−1^)	(µS cm^−1^)			(mg L^−1^)
Son Morell	20	580	2.1	Ephemeral (days-weeks)	1.7	1.03	0.5	0.8	14.1	252	8.29	6.8	3.7
Curniola	150	769	173	Intermittent (>10 months)	0.36	0.41	0.4	—	10.5	171	6.86	5.4	3.5
Verda d’Algaiarens	140	863	1.1	Intermittent (8–10 months)	1.32	0.05	2.9	0.3	9.1	347	6.72	23.6	22.7
Torrellafuda	165	2448	107	Intermittent (7–8 months)	2.61	1.36	1.2	1.3	14.6	213	8.55	10.2	11.9
Mal Lloc	155	832	12.3	Intermittent (10–11 months)	2.52	0.43	1.4	0.9	6.6	331	6.76	33.7	28.7
Verda des compte	91	1045	1.2	Ephemeral (weeks)	—	—	—	—	—	—	—	8.5	—
Armaris	58	4369	6.1	Intermittent (9–10 months)	1.08	0.04	0.8	1.8	14.7	1201	9.85	11.5	15.1
Sa Mesquida	103	313	0.2	Intermittent (9–10 months)	0.67	0.02	2.2	3.7	7.7	1311	7.72	10.1	12.4
Cap Negre	49	351	0.3	Ephemeral (weeks)	0.72	0.05	1.5	0.9	11.8	298	8.04	2.1	12.1
Marina Curniola	55	149	3.1	Ephemeral (weeks)	0.71	0.04	1.1	0.8	17.6	113	9.6	8.5	5.6

TN: total nitrogen, TP: total phosphorus, Chl-a: Chlorophyll a, Alk: alkalinity, EC: electrical conductivity, LOI: Loss on Ignition, DOC: Dissolved Organic Carbon. The duration of the wet phase refers to the time period during which the ponds present a water layer of any extension.

## Results

### Magnitude and variability of CO_2_ and CH_4_ fluxes

**Figure 1:**
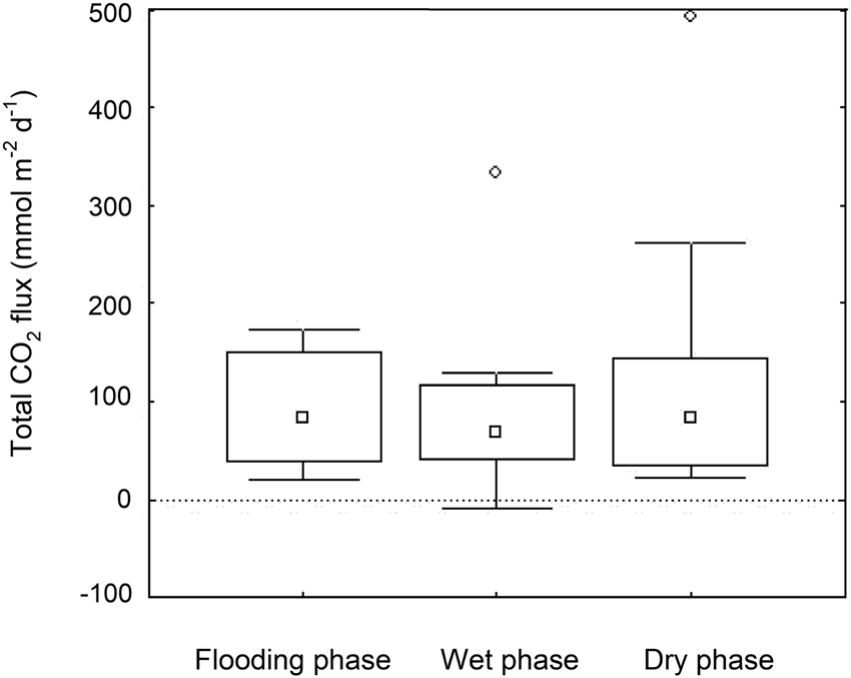
Total CO_2_ flux (T-FCO_2_) measured during the flooding, wet and dry phases of temporary ponds. The median (squares), non-outlier range and outliers (whiskers and white dots), and 25^th^ and 75^th^ percentiles (box) are shown for the 10 studied ponds.

The ponds were net emitters of CO_2_ to the atmosphere irrespective of the hydrological phase, with a mean total flux (i.e. spatially integrated flux including all habitats, T-FCO_2_) of 107.8 ± 103.9 mmol m^−2^ d^−1^ (±standard deviation; n = 30; Table 2; Fig. 1). We observed small negative T-FCO_2_ only on one occasion (Table 2). There was high variability in T-FCO_2_ between ponds and between phases (range 15.7 to 492.5 mmol m^−2^ d^−1^). The maximum T-FCO_2_ was measured during the dry phase (Table 2; Fig. 1), although we did not observe any significant seasonal pattern in T-FCO_2_ along the hydrological cycle (ANOVA between phases, F = 0.34, p > 0.05; Fig. 1). In contrast, CH_4_ fluxes were of minor importance and only detectable on a few occasions in dystrophic systems (Tables 1 and 2; detection limit estimated to be 0.04 mmol m^−2^ d^−1^).

**Table 2:** Total CO_2_ (T-FCO_2_) and CH_4_ (T-FCH_4_) fluxes from the temporary ponds along different phases of their hydrological cycle.

Pond	Flooding phase		Wet phase		Dry phase	
	T-FCO_2_	T-FCH_4_	T-FCO_2_	T-FCH_4_	T-FCO_2_	T-FCH_4_
Son Morell	98.5 ± 1.3	*n.d*.	77.7 ± 11.2	*n.d*.	28.3 ± 4.5	*n.d*.
Curniola	70.4 ± 16.2	*n.d*.	15.7 ± 1.7	*n.d*.	33.8 ± 21.3	*n.d*.
Verda d’Algaiarens	151.1 ± 5.3	*n.d*.	43.3 ± 6.7	0.1 ± 0.3	44.4 ± 5.7	*n.d*.
Torrellafuda	61.6 ± 7.7	*n.d*.	333.1 ± 20.7	*n.d*.	119.7 ± 8.3	*n.d*.
Mal Lloc	172.4 ± 10.5	*n.d*.	129.2 ± 2.6	1.1 ± 0.3	262.3 ± 19.2	0.1 ± 0.1
Verda des Comte	23.6 ± 7.5	*n.d*.	117.2 ± 26.6	*n.d*.	75.3 ± 36.0	*n.d*.
Armaris	166.7 ± 6.7	*n.d*.	−8.5 ± 4.6	*n.d*.	22.9 ± 3.4	0.1 ± 0.3
Sa Mesquida	20.1 ± 2.7	*n.d*.	40.9 ± 5.9	*n.d*.	492.5 ± 10.5	*n.d*.
Cap Negre	39.5 ± 3.4	*n.d*.	59.6 ± 20.2	*n.d*.	89.3 ± 4.7	0.1 ± 0.1
Marina Curniola	94.8 ± 7.0	*n.d*.	98.4 ± 7.2	*n.d*.	144.9 ± 43.4	*n.d*.

Data corresponds to the mean and standard deviation for each pond (mmol m^−2^ d^−1^). *n.d.: not detected*.

**Figure 2:**
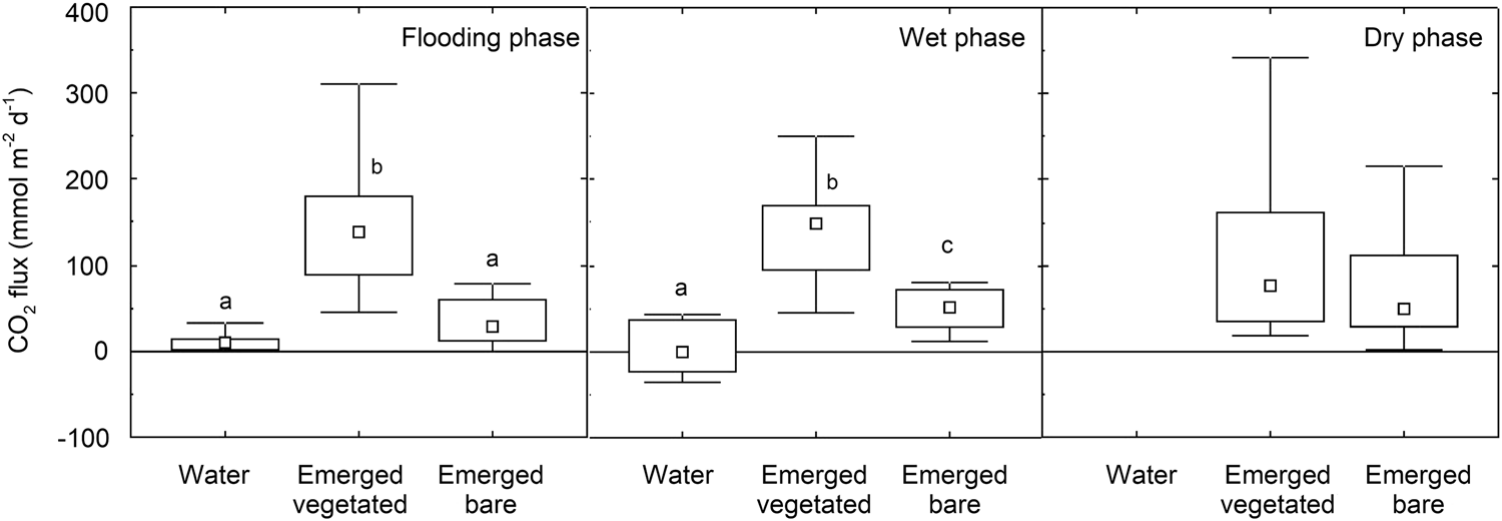
CO_2_ fluxes from the different habitats of the ponds for the three hydrological phases. Letters indicate significant differences between habitats in each season (Tukey post-hoc tests, p < 0.01). Symbols as in Fig. 1.

**Figure 3:**
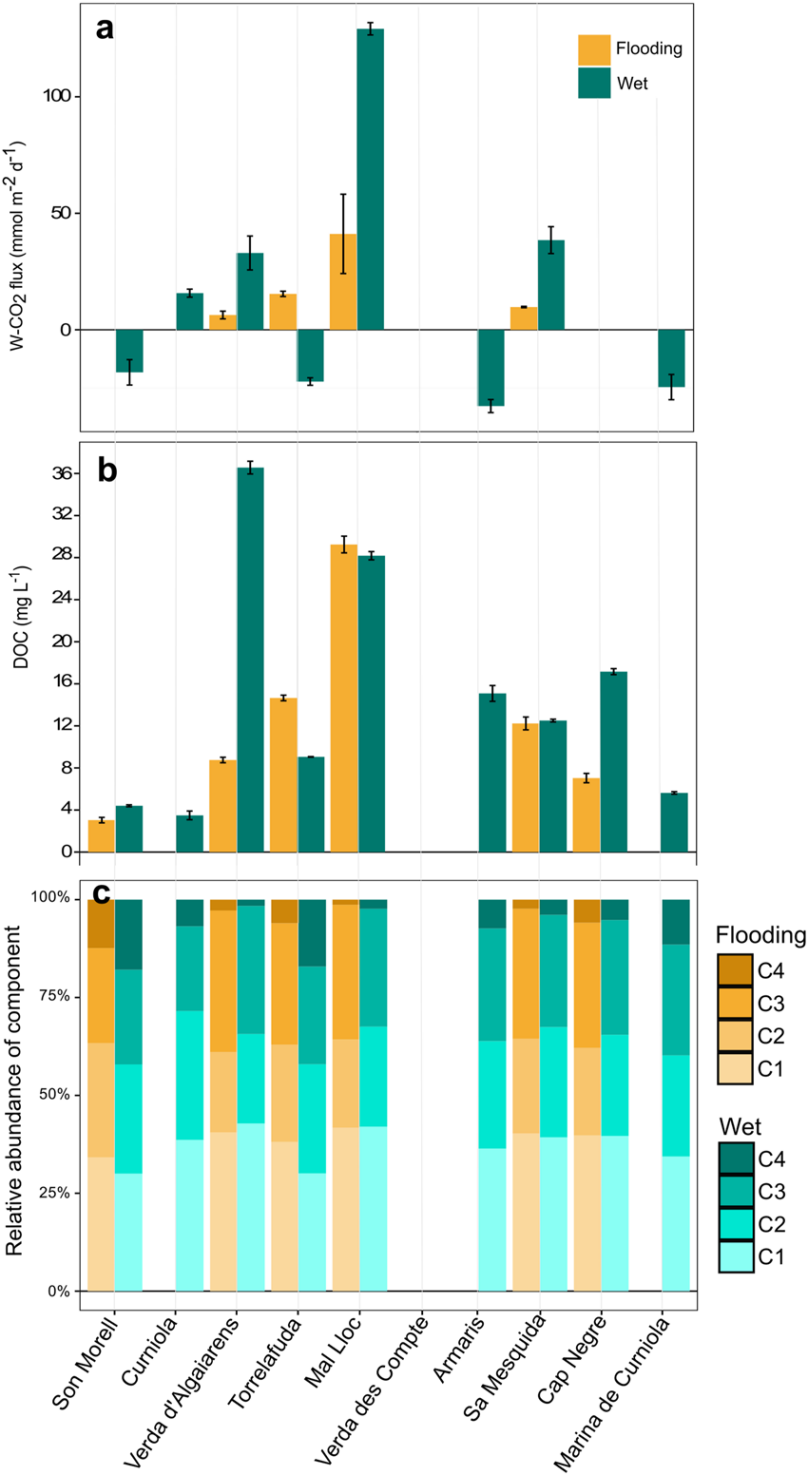
CO_2_ fluxes and dissolved organic matter quantity and composition in the water of the ponds for the flooding and wet phases. (**a**) CO_2_ flux from the inundated habitats of the ponds (W-FCO_2_); (**b**) DOC concentration; (**c**) PARAFAC extracted components of DOM. The ponds Curniola, Verda des Comte, and Marina de Curniola were completely dry during the flooding phase. The ponds Verda des Comte and Cap Negre were completely dry during the wet phase. Bars and lines denote mean and standard deviation.

The different habitats of the ponds (inundated, emerged-vegetated and emerged-bare) were all active in terms of CO_2_ fluxes (Fig. 2). The minor role of inundated habitats contrasted with the high efflux measured in emerged habitats in all phases of the hydrological cycle. Emerged habitats showed significantly higher rates than inundated habitats when present (ANOVA F = 68.9 and F = 32.2 for the flooding and wet phases, respectively, p < 0.01). Within the emerged areas of the ponds, the emerged-vegetated sediments showed higher effluxes than the emerged-bare habitats both in the flooding and wet phases (Tukey test > 0.01). Differences between emerged-vegetated and -bare habitats were indistinguishable in the dry phase (Fig. 2). Interestingly, in four ponds the inundated habitats had negative fluxes (i.e. invasion of CO_2_ from the atmosphere into the water) during the wet phase (Fig. 3a) although these fluxes were compensated by the positive fluxes from the other habitats. In contrast, during the flooding phase, all inundated habitats were emitters of CO_2_ to the atmosphere.

### **Drivers of CO**_**2**_**fluxes**

Concentration and composition of organic matter was determined in the different habitats to identify the drivers of the CO_2_ fluxes. Dissolved organic carbon (DOC) concentration in the water column ranged from 3 to 36.6 mg L^−1^, with high variability between ponds (Fig. 3b). Dissolved organic matter (DOM) composition was assessed by optical spectroscopy. A Parallel Factor Analysis (PARAFAC) modelling of the excitation emission matrices of 99 samples revealed 4 independent components, (C1 – C4; *see* Supplementary Information S2). Components C1 (Ex: 250 (340), Em: 450) and C3 (Ex: <250 Em: 510) represent humic-like ubiquitous components related to terrestrial sources. Component C2 (Ex: 250 (300), Em: 400) is associated with humic materials having suffered some degree of microbial processing^32^. Finally, component C4 (Ex 280, Em: 340) presents protein-like characteristics similar to tryptophan^33^.

**Figure 4:**
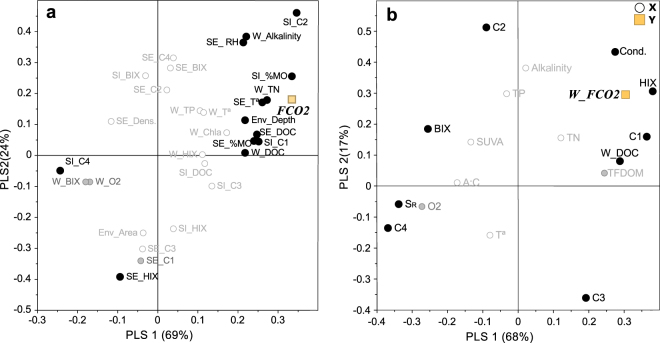
Loading plots of the PLS analyses of a) the total CO_2_ fluxes (T-FCO_2_), and b) the CO_2_ fluxes from the inundated habitats of the ponds (W-FCO_2_). The Y-variables are shown in squares. The X-variables are classified according to their VIPs (influence on the Y-variable, *see* Tables S3.1 and S3.2): highly influential (black circles), moderately influential (grey circles), and less influential (white circles). Abbreviations stand for variables measured in **W** (water), **SI** (sediments-inundated), **SE** (sediments-emerged) and **Env** (ecosystem characterization), and correspond to: **%MO:** Sediment organic matter content (LOI, %); **DOC:** DOC (mgC L^−1^); **TN:** Total nitrogen (mgN L^−1^); **TP:** Total phosphorous (µgP L^−1^); **Chla:** Chlorophyll – a (µg L^−1^); **O2:** oxygen concentration (mg L^−1^); **RH:** Relative humidity sediment (%); **Dens.:** Sediment density (g L^−1^); **Cond.:** Sediment conductivity; **Depth:** Pond depth (cm); **Area:** Area (m^2^); **Tª:** Temperature (°C); **C1:** Fluorescent component 1 (%); **C2:** Fluorescent component 2 (%); **C3:** Fluorescent component 3 (%); **C4:** Fluorescent component 4 (%); **HIX:** Humification Index (dimensionless); **BIX:** Biological Index (dimensionless).

A PLS regression model describing the response of T-FCO_2_ (i.e. spatially integrated flux; Fig. 4a; Table S3.1) extracted two significant components that explained 92% of variance (R2Y = 0.92) with high model predictability (Q2cum = 0.64). The T-FCO_2_ flux (Y-variable) was predicted by the amount and composition of organic matter across the different habitats (Fig. 4a). Specifically, the humic-like component C2 in the inundated sediment was the most important predictor, followed by organic matter concentrations in the inundated and emerged sediments, and, with slightly lower influence, DOC in water (Table S3.1). Other important predictors were total nitrogen and alkalinity (in the water column when present), temperature and humidity (in the emerged sediments), and the protein-like component C4 (in the inundated sediments), negatively correlated with FCO_2_. It was impossible to fit a PLS model to explain FCO_2_ in the dry habitats. The PLS model for FCO_2_ from inundated habitats (i.e. flux from water, W-FCO_2_; Fig. 4b; Table S3.2) extracted two significant components explaining 86% of variance (R2Y = 0.87) and presented high predictability power (Q2cum = 0.67). W-FCO_2_ was related to descriptors of DOC composition, such as the humification index (HIX), the PARAFAC components, and the spectral slope (S_R_). Autochthonous or microbial-like DOM descriptors such as the biological index (BIX), C4 or S_R_ as well as oxygen concentration were well correlated with each other and negatively correlated with W-FCO_2_. Indicators of humic-like materials such as HIX or component C1 appeared to be associated with DOC concentrations and were positively related with W-FCO_2_. Accordingly, systems with higher DOC concentrations presented also higher contribution to DOM composition of humic-like components C1 and C3 and, in most cases, these humic-like compounds were lower in the flooding than the wet phase (Fig. 3b and c). In contrast, the relative contribution of components related to biological activity, C2 and C4, was higher in systems with lower DOC concentrations. In most cases this corresponded to negative W-FCO_2_ during the flooded phase.

Analysis of the bacterial community in the sediments revealed 512,136 reads that clustered into 8,621 OTUs distributed unevenly across 8 studied sites (samples of ponds Verda des Comte and Verda d’Algaiarens were lost during procedure; Suppl. Table S4). As a general trend, bacterial communities were dominated by Actinobacteria and Alphaproteobacteria, although some communities also showed a high prevalence of sequences affiliated to Chloroflexi and Firmicutes (Suppl. Fig. S4a). Although the inference of functional properties from phylogeny is always risky^34^, the vast majority of sequences were affiliated with genera encompassing heterotrophic representatives, either aerobic or facultative anaerobes (data not shown). Remarkably, the relative abundance of sequences affiliated to methanotrophic bacteria was especially high in Cap Negre, Torrellafuda and Curniola (>3% of total bacterial reads, Suppl. Fig. S4b). The presence of potential methanotrophs was only negligible in Armaris. For Archaea, the 447,892 raw sequences clustered into 240 OTUs, which were mostly affiliated with members of the Soil Crenarchaeota Group (Thaumarchaeota) (Suppl. Fig. S4c). Methanogenic archaea were only relevant in terms of their relative abundance in Curniola, where they accounted for 28.4% of total archaeal reads.

**Figure 5:**
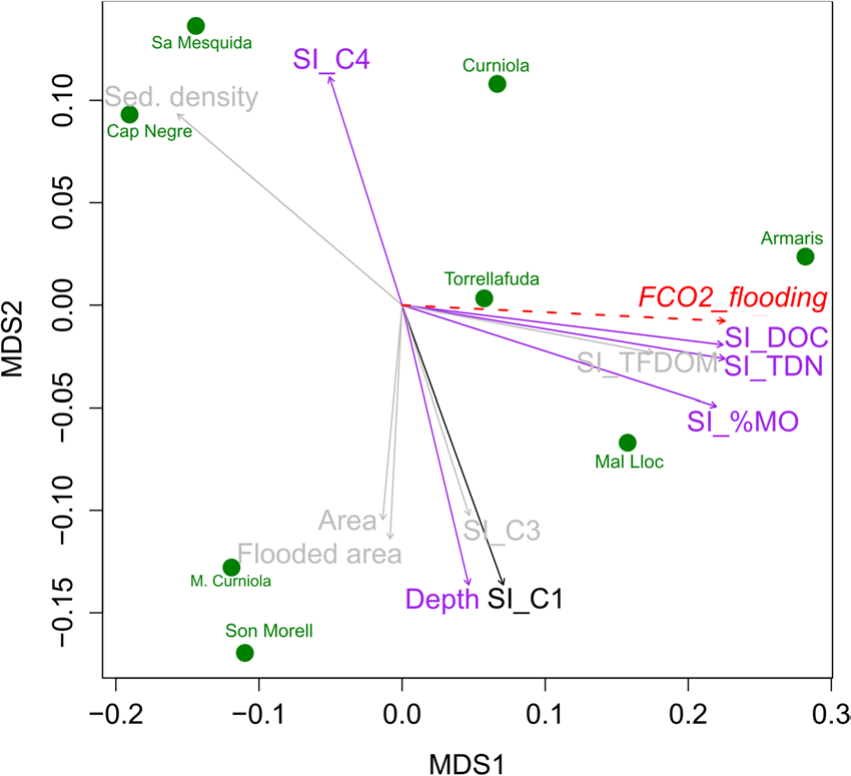
Non- metric dimensional scaling (NMDS) plot of the weighted UniFrac distances of the bacterial community data in 8 of the studied temporary ponds during the flooding phase. The arrows represent those inundated sediment and water descriptors that were significantly related with the ordination (p-value < 0.001 in black; p-value < 0.05 in purple and p-value < 0.01 in grey). The total FCO_2_ during the flooding phase was not significantly related (p - value = 0.1) but has been plotted to illustrate the relationship with the organic C descriptors previously assessed in Figs 3 and 4.

The bacterial community composition (BCC) appeared to be linked to the presence and composition of organic matter in the sediments. The first dimension of the non- metric multidimensional scaling (NMDS, Fig. 5) was significantly related to the amount of organic matter in the sediments (negative values associated with higher sediment density). The second axis was related to DOM composition, with a gradient between the protein like component C4 and the humic-like component C1 (Fig. 5). Similar links between these physicochemical descriptors and bacterial abundance could not be found.

## Discussion

All the temporary ponds studied were net emitters of CO_2_ to the atmosphere. The emissions reported here (mean 107.8 mmol m^−2^ d^−1^, range 15.7 to 492.5 mmol m^−2^ d^−1^) were much higher than a recent global estimate for small (<0.001 km^2^) permanent ponds (mean 35.2 ± 5.2 standard error;^10^) and in the same range described for intermittent rivers^28^ and temporary wetlands^35^. The high variability in the fluxes between and within ponds prevented the identification of clear temporal patterns, but depicted the ponds as net emitters of CO_2_ regardless of the hydrological phase, as shown by comparable emission rates during the flooding, wet and dry phases (Fig. 1). This is a relevant finding that supports recent studies presenting temporary aquatic systems as extremely active biogeochemical sites during their dry phases^11,29^.

Interestingly, the emerged habitats were key areas contributing to the total CO_2_ emissions of the ponds. Emerged habitats contributed substantially to the total surface area of the ponds not only during the dry phase but also in both flooding and wet phases (80% and 51% on average, respectively, Table S1). More importantly, emerged habitats showed higher emissions per unit area than the inundated habitats, hosting fluxes generally above 50–150 mmol CO_2_ m^−2^ d^−1^depending on the presence or absence of vegetation (Fig. 2). This agrees with recent findings reporting high CO_2_ emissions (well above 100 mmol CO_2_ m^−2^ d^−1^) from dry sediments in rivers and reservoirs^28–30^. This is much higher than the literature values for C emissions from lakes, reservoirs, and permanent ponds^4–10^ (18–55 mmol m^−2^ day^−1^). Higher emissions from emerged than from inundated habitats may be due to the physical limitation of gas diffusion under inundated conditions^28,36^ or boosted microbial activity in dry conditions^37,38^. Higher oxygen availability under dry conditions may enhance the release of labile organic materials through microbial cell lysis and physical processes^39^. However, microbial activity may not behave linearly in relation to drying, as enzyme activity is considerably decreased if water content drops below certain levels during complete desiccation^30,40^. In any case, the interplay between drying-rewetting events, sediment water content, microbial activity, and gas diffusivity is complex, as is demonstrated by the extensive literature on this topic in wetlands, soils, and more recently dry river beds^40–42^. Indeed, in order to achieve a complete understanding of C mineralization and gas fluxes in the dry sediments of aquatic ecosystems, it must be considered that 1) short-term CO_2_ pulses after drying-rewetting events may hide a substantial part of total C annual fluxes from these environments^39^ and 2) biological activity is the most plausible origin for the CO_2_ emissions from dry areas, but other processes such as carbonate reactions with the system or photochemical degradation of organic matter may also contribute to CO_2_ efflux^43–45^.

Within the emerged habitats, vegetated sediments showed higher emissions than bare sediments despite the large variability in vegetation types across ponds, which included perennial macrophytes, small annual helophytes and hydrophytes^46^. The higher emission in vegetated habitats can be related to either direct CO_2_ production by root respiration (i.e., autotropic respiration) or to indirect changes in sediment texture and porosity facilitating gas diffusion^47,48^. Unfortunately, our results do not allow for establishing complete C budgets in the ponds, since primary production by the vegetation was not measured.

In contrast to CO_2_ fluxes, CH_4_ fluxes were of minor importance across all the ponds and habitats and exclusive to the inundated habitats of dystrophic systems. Although this observation is contrary to the presence of methanogenic lineages in some of the ponds, the co-occurrence of methanotrophic bacteria in the sediments provides a plausible explanation for the low CH_4_ emissions measured. Moreover, although CH_4_ production has been detected in arid soils, production rates are usually very slow when oxygen is present^49^ and soil humidity low^50^. Thus, the conditions in the emerged sediments studied explain the low CH_4_ emissions detected in these habitats despite the microbial methanogenic potential of the community. Also, it is likely that episodic high CH_4_ fluxes were not captured with our sampling design. In an Amazonian floodplain lake, relevant CH_4_ emissions were only observed during the first few days after drying and later CH_4_ emissions from dry sediments were negligible^51^. The low CH_4_ emissions measured in temporary ponds here contrasts with the high CH_4_ emissions reported for permanent ponds and small lakes, where ebullitive CH_4_ emissions can be significant^3,10^.

Another relevant finding of this study is the divergent CO_2_ fluxes of inundated and emerged habitats. We showed that fluxes from inundated habitats were not only lower than those from emerged habitats but they were negative (i.e., acting as C sinks; Fig. 2) during some phases. This observation contrasts with the predominant role of the ponds as CO_2_ emitters when all habitats were integrated. However, our emission rates from dry areas are likely a maximum estimate because we did not measure primary production by vegetation. Taking into account the typical hydroperiod of these temporary ponds (Table 1) and the areas of the different habitats (Table S1), the inundated habitats are unlikely to dominate the total pond area on an annual basis. Accordingly, ponds likely act very rarely as net C sinks. In this sense, even though this dataset captures spatial flux variability by sampling all pond habitats, the discrete temporal approach used prevents thorough estimates of annual budgets. Such estimates would require continuous monitoring of fluxes along a hydrological year.

The contrasting pattern between inundated vs emerged habitats undermines traditional approaches of temporary pond biogeochemistry, where those habitats not inundated or those phases without water are typically not considered^25,26,31^. Our results highlight the need for spatially and temporally inclusive approaches to characterise C biogeochemistry in temporary systems, as has been recently pointed out in other temporary aquatic ecosystems such as rivers^11,28,29^ or reservoirs^30^.

The total (T-FCO_2_) and the water (W-FCO_2_) fluxes were predicted by descriptors of organic matter composition and quantity that segregated ponds with contrasting trophic state (Figs 3 and 4). On the one hand, ponds with higher FCO_2_ showed higher concentrations of humic organic matter in the water and sediments. FCO_2_ has been previously related to organic C concentration in water^52,53^ and dry river beds^29^ as well as with humic substances^5^, typically in boreal or deciduous forest areas. W-FCO_2_ is traditionally related to the oxidation of terrestrial substances within the water column. Indeed, the ponds found to have positive W-FCO_2_ throughout the campaigns had a notably dystrophic character (Fig. 3; Table 1) with elevated concentrations and coloured DOC. However, these coloured lignin-derived compounds are likely to be related with the standing submerged^54^ and surrounding riparian vegetation, as no relationship was previously found with catchment characteristics^17^. The CO_2_ fluxes from emerged habitats could not be explained by the measured variables in an independent PLS model. However, the variables selected in the PLS model for the T-FCO_2_, which includes the emissions from the emerged habitats, are in agreement with the drivers of CO_2_ fluxes from dry sediments shown elsewhere^29^, such as organic matter concentration or relative humidity (Fig. 4).

On the other hand, we found that W-FCO_2_ was related to protein-like DOM from autochthonous sources. The switch from positive to negative W-FCO_2_ between the two phases with water, which occurred in parallel with a relative increase of the protein-like component, points towards a higher influence of the *in-situ* production in some systems. However, DOM sources showed no change between phases in the dystrophic systems (Fig. 3). Enhanced contributions of DOM from autochthonous sources are frequently related to the release of more bioavailable compounds^55^. Therefore, increased respiration and FCO_2_ might be expected. However, if this autochthonous production is due to autotrophic activity, it might lower or even make the final flux of CO_2_ negative. Accordingly, ponds with a negative W-FCO2 during the flooded phase (Fig. 3) were mostly shallow. In these shallow systems the role of aquatic primary producers is likely to manifest as influx of CO_2_. In addition, evidence of occasional livestock presence in the surroundings and the existence of biological crusts were observed in these systems. These can also be important sources of protein-like DOM^56^.

The role of organic C becomes even more apparent when we explore links with bacterial community composition (BCC). The amount and especially the composition of organic matter in the sediments explained differences in BCC among sites (Fig. 5). Within these two axes of variability, T-FCO_2_ is related to the extractable amount of DOM from the sediments and total fluorescence, which mostly relates to the presence of humic compounds. It is challenging to link BCC to an integrative biogeochemical response such as FCO_2_ since respiration is a metabolic response for which community members are functionally redundant. However, the BCC of sites rich in sediment organic matter and humic substances (sites with high FCO_2_ fluxes) diverged from those sites with protein-like DOM compounds (sites with lower FCO_2_ fluxes). Thus, the ability to degrade particular organic compounds does seem to be linked to specific communities^57^. This fact might explain why community composition is more important to explain DOM composition and ultimately FCO_2_ in the studied ponds than bacterial abundance, as found in Morrissey *et al*.^58^.

## Conclusions and Perspectives

Small ponds are still not included in most large-scale inland waters inventories^20^. Moreover, if these small systems undergo a dry phase during the hydrological year, they are almost totally excluded from biogeochemical studies. Here, we show that these temporary pond systems and their dry habitats in particular (i.e. emerged sediments) contribute significantly to CO_2_ emissions. Moreover, the dual role of inundated habitats acting both as sink and source of CO_2_ depending on the hydrological phase illustrates the partial picture that we can get if studying exclusively the inundated phase of aquatic systems, as traditionally done^4^.

Changes in the hydroperiod of these ponds might either increase or decrease the extension of these dry habitats, resulting in changes in the magnitude of these fluxes. Hot moments of high emissions as shown for periodically dry lake sediments^51^ may significantly contribute to the C budget of these systems. Finally, organic matter composition and microbial community composition merit further study, as they provide mechanistic understanding on the gas fluxes controls. Understanding the interplay between physical and biological processes will contribute key information to predict the occurrence of gas effluxes not only in temporary ponds but in all types of temporary inland water ecosystems.

## Methods

### Study sites and sampling design

We studied 10 temporary ponds located on the island of Menorca (Balearic Islands, Western Mediterranean) (Table 1). The climate is Mediterranean with a dry and hot summer period and mean monthly temperatures ranging from 10 °C in January to 25 °C in August. The studied systems were selected from a detailed inventory of temporary ponds on the island aimed at covering the widest possible spectrum of physicochemical, geomorphologic and hydrological properties^46^. Ephemeral ponds located in rocky basins were discarded. Main land uses include pine forests, Mediterranean shrublands, and, in some catchments, dry and irrigated croplands. A previous study showed no significant effect of the land cover or catchment lithology on CO_2_ fluxes^17^. Three samplings were performed during the hydrological year of 2014 (from September 2013 to August 2014) to characterise contrasting hydrological situations. A first sampling was performed on November 2013 to characterise the *flooding phase* of the ponds, with a cumulative rainfall of 130 mm since the end of the previous dry phase (September 2013; Suppl. Figure 1). A second sampling on March 2014 represented the *wet phase* (cumulative rainfall of 454 mm). A final sampling on August 2014 characterised the *dry phase* (all ponds were entirely dry) and took place with a cumulative rainfall of 548 mm and after 63 days without any rainfall.

Each pond was divided in three different habitats including inundated and non-inundated areas. The *inundated* habitats were defined as those areas with a water layer during sampling. The *emerged-vegetated* habitats were defined as those non-inundated areas with some vascular plants present. The *emerged-bare* habitats were defined as those non-inundated areas with bare sediments or just biological crusts on them. All three habitats were sampled if present. On each sampling campaign the surface of each habitat was measured *in-situ* and then digitized using aerial photography and the cartographic information in Fraga *et al*.^46^.

### Gas flux measurements

To guarantee the comparability of the flux measurements, the same method was used in all habitats of the ponds. Direct measures of the flux of CO_2_ (FCO_2_) and of CH_4_ (FCH_4_) were performed *in situ* with a chamber method^59^ and restricted as much as possible to the central time of the day. Three randomly distributed replicate FCO_2_ measurements were performed in each habitat using opaque enclosed chambers connected to an infrared gas analyser (IRGA EGM-4, PP-Systems, Amesbury, USA). The CO_2_ concentration inside the chamber was monitored every 4.8 s, with an accuracy of 1%. The floating chamber for air-water flux measurements had a surface area of 0.194 m^2^ and a volume of 27.1 dm^3^. The chamber for flux measurements in emerged sediments had a surface area of 0.0078 m^2^ and a volume of 1.171 dm^3^ (SRC-1, PP-Systems, Amesbury, USA). Flux measurements in emerged sediments refer to CO_2_ emissions from the substrate and thus integrate both sediment and root respiration but no C fixation by vegetation. The flux measurements lasted until at least 10 µatm of change in CO_2_ were reached, with a maximum duration of 600 s in water (minimum of 300 s) and 300 s in emerged sediments (minimum of 120 s). Fluxes were determined by linear regression between the CO_2_ concentration in the chamber and time (R^2^ > 0.9), correcting for temperature and atmospheric pressure^60^.

FCH_4_ was determined by duplicate in each habitat with the same chambers described above and a duration of between 25 and 60 min. Initial and final gas samples from the chambers were taken with a 30 ml polypropylene syringe, mixing the gas inside the chamber prior to sampling. The gas samples were preserved in pre-evacuated vials (Exetainers 339 W, Labco Lim. Lampeter, UK) and analysed for CH_4_ concentration within 7 weeks in a gas chromatograph equipped with a flame ionization detector (SRI 8610, SRI Instruments, Torrance U.S.A.). A 4 ppm CH_4_ in N_2_ standard gas mixture was used for calibration of the gas chromatograph.

The total flux of the ponds (T-FCO_2_ and T-FCH_4_) was obtained by weighing the mean specific areal flux in each habitat by the surface area of each habitat. This flux does not include C fixation from emerged vegetation, which would require different experimental approaches to measure. All fluxes were expressed in mmol m^−2^ d^−1^, with the convention that positive values correspond to effluxes to the atmosphere and negative values to influxes into the ponds.

### Characterisation of the ponds

#### Water properties

We used portable probes to measure *in situ* water temperature, conductivity, pH (WTW, Germany), and dissolved oxygen (DO; YSI ProODO Handheld, Ohio, USA). Triplicate water samples were taken at each inundated pond during every campaign. We analysed total Phosphorus (TP) and nitrogen (TN)^61^ and alkalinity^62^ on unfiltered samples. Chlorophyll a concentration was determined by the trichromatric method after 90% Acetone extraction^63^. DOC and TDN concentrations were determined in a Shimadzu TOC-VCS with a coupled TN analyzer unit on previously filtered (pre-combusted and pre-rinsed GF/F filters (Whatman, GE Healthcare, UK)) and samples acidified to pH 2–3 with HCl 2 M. Samples for DOM characterization were filtered through pre-combusted and pre-rinsed GF/F filters (Whatman, GE Healthcare, UK) and stored cold at 4 °C in glass vials until analysis within the same week.

#### Sediment properties

Sediment temperature and humidity were measured with a portable soil probe in the locations where flux measurements were done (Decagon ECH2O 10HS, Pullman, USA). Sediment pH and conductivity (WTW, Germany) were measured in a 1:1 sediment: Milli-Q mixture^64^. In these same spots we collected sediment samples (0–5 cm depth) that were frozen until further analysis. Once in the lab, the water and organic content of sediments was determined by drying and subsequent loss on ignition. Water extractable organic matter (WEOM) was extracted from freeze-dried and grounded sediments by shaking them with Milli-Q (sediment:water ratio of 1:10) in a dark incubator for 48 h at 4 °C. Then the extracts were centrifuged (10 min, 4,500 rpm) and filtered through GF/F and 0.45 µm nylon membrane (Whatman, GE Healthcare, UK) Milli-Q pre-rinsed filters^65^. The raw DOC concentration of the extracts was measured and diluted to a stock concentration of 10 mg L^−1^ C. Sediments were considered stable across campaigns, and therefore a single characterization was done for their organic matter content.

#### DOM and WEOM characterization

UV–Vis absorbance spectra (200–800 nm) were obtained in a Shimadzu UV- 1700 spectrophotometer using a 1 cm quartz cuvette and allowing samples to warm up to room temperature prior to analysis. The absorption coefficients at wavelength λ (a _λ_, m^−1^) were determined using the expression: a_λ_ = 2.303 A_λ_ /l, where A_λ_ is decadal absorbance and l is path length in metres. The slopes (S) of the spectra were obtained by non-linear fitting of the exponential curve a_λ_ = a_λ0_ e ^S(λ^_0_^−λ)^^66^, where λ_0_ is a reference wavelength. The slope ratio S_R_, related with molecular weight, was calculated as the ratio of slopes between the wavelengths in subscripts: S_275–295_ /S_350–400_^67^. SUVA (L mg C^−1^ m^−1^), an indicator of aromaticity was calculated as the absorbance at 254 nm normalized by DOM concentration^68^.

Fluorescence excitation - emission matrices (EEM) were obtained with a fluorescence spectrophotometer (Shimadzu RF-5301PC). Excitation wavelengths ranged from 240 nm to 400 nm at intervals of 10 nm, and emission wavelengths ranged from 270 nm to 630 nm at increments of 1 nm. A Milli-Q blank was subtracted from each spectra to account for Raman scattering. The area underneath the water Raman scan was used to normalize all sample intensities. Correction-factors supplied by the manufacturer were used to correct for instrument-specific biases. Spectra were corrected for the inner filter effect using the absorbance-based approach^69^. All the corrections were applied using the FDOM correct toolbox for MATLAB (Mathworks, Natick, MA, USA) following Murphy *et al*.,^70^. Finally, we determined the biological index (BIX) according to Huguet *et al*.^71^ and the humification index (HIX), described in Ohno^72^.

Parallel Factor Analysis (PARAFAC) was used to identify the main components of DOM^73^ over the analysed 99 EEMs (incuding DOM and WEOM). This analysis was performed using the DrEEM toolbox for MATLAB (Mathworks, Natick, MA, USA) according to Murphy *et al*.^74^. Scatter peaks and outliers were removed, and samples were normalized to the total fluorescence of each prior to fitting a PARAFAC model. The appropriate number of components was determined by visual inspection of the residual fluorescence and of the components behaviour as organic fluorophores^74^. A 4 components model was then validated by split-half analysis and random initialization with 10 iterations (Supplementary Figure 2). To qualify the identified components we queried them (TCC = 96%) in the OpenFluor data base (http://www.openfluor.org75; January 2016). This online tool allows for testing them against identified and characterized PARAFAC models in previous DOM studies (*see* Supplementary Information S2 for further details).

#### Microbial community: DNA extraction and high-throughput sequencing

A two-gram subsample of each sediment sample was subjected to DNA extraction using the FastDNA Spin Kit for Soil (MP Biomedicals) according to manufacturer’s instructions. Concentration of DNA in each extract was then determined fluorimetrically using QUBIT®2.0 Fluorometer (Invitrogen Molecular probes Inc., Oslo, Norway). DNA extracts were analysed through MiSeq PE2x250 Illumina chemistry at the Research and Testing Laboratory (RTL Lubbock, TX, USA). Genomic DNA from sedimentary communities was used as a template in PCR reactions using specific primers targeting the V1-V2 and V4-V5 hypervariable regions of the bacterial and archaeal 16 S rRNA genes, respectively (Supplementary Information S5). Both primer pairs were complemented with Illumina-adapters and sample-specific barcodes. Raw sequence datasets were quality filtered, chimera-checked and clustered into Operational Taxonomic Units (OTUs, 97% cutoff) using QIIME^76^. Construction of OTU table and downstream analysis were also conducted in QIIME. Technical details about the sequence processing pipeline are provided in the Supplementary Information (Supplementary Methods S5). All raw bacterial and archaeal DNA sequences retrieved in this study are publicly available through the Sequence Read Archive (SRA) database under accession SRP126916 (https://www.ncbi.nlm.nih.gov/sra).

### Data analysis

Differences in fluxes between hydrological phases were tested with one-way analysis of variance (ANOVA). Differences in flux between the habitats of the ponds (i.e. inundated, emerged vegetated sediments and emerged bare sediments) in each phase were tested with ANOVA followed by Tukey post-hoc comparison. All variables were log transformed prior to analysis. These statistical analyses were performed in STATISTICA v9 software and were only applied on CO_2_ fluxes because of the low number of detectable CH_4_ fluxes.

To identify the main drivers of total CO_2_ flux as well as those in water and in emerged sediments, we performed partial least squares projections to latent structures (PLS). PLS is used to find relationships between a matrix of explanatory variables X and a matrix of response variables Y. The PLS model performance is expressed as R2Y (explained variance) and Q2, a measure of the predictive power of the model, assessed by cross-validation. We permuted 100 times the response variable, to estimate the statistical significance of the predicted power. To summarize the relative importance of the predictors for explaining Y, we used the variable influence on the projection (VIP^77^). VIP-values larger than 1 indicate the most influential predictors, between 1 and 0.8 moderately important, and <0.8 less influential X-variables. Skewed variables were log-transformed prior to analysis. PLS analysis of data matrices included the physico-chemical descriptors of the whole ecosystem or each specific habitat. The annual mean value of all variables was considered for this analysis. The PLS analysis was performed in SIMCA-P (Umetrics AB, Umeå, Sweden).

Non-metric dimensional scaling (NMDS) was used to ordinate the ponds according to their bacterial community composition using the weighted UniFrac distances and the *metaMDS* function. The matrix used for the PLS was fitted to the NMDS as linear vectors using the *envfit* function. All these functions are available in the *vegan* package^78^ for R^79^.

### Data availability

Data supporting these findings is available in the text or the supporting information.

## Electronic supplementary material

Supplementary information
