# Supplementary material for: Dry habitats sustain high CO2 emissions from temporary ponds across seasons
Source: Sci Rep. 2018 Feb 14;8:3015. doi: 10.1038/s41598-018-20969-y (PMC5813041; doi:10.1038/s41598-018-20969-y)
Supplement: Supplementary file 1 — Supplementary information [file 41598_2018_20969_MOESM1_ESM.pdf]

Supplementary information

Dry habitats sustain high CO2 emissions from temporary ponds across seasons

Biel Obrador[1,*], Daniel von Schiller[2,3], Rafael Marcé[3], Lluís Gómez-Gener[1,4], Matthias Koschorreck[5], Carles Borrego[2, 6], Núria Catalán [1, 2, 7,*]

Affiliations

[1] Department of Evolutionary Biology, Ecology and Environmental Sciences, University of Barcelona, Av. Diagonal 643, 08028 Barcelona, Spain

[2] Catalan Institute for Water Research (ICRA), Scientific and Technological Park of the University of Girona, Emili Grahit 101, 17003 Girona, Spain.

[3] Department of Plant Biology and Ecology, Faculty of Science and Technology, University of the Basque Country, Apdo. 644, 48080 Bilbao, Spain.

[4] Department of Ecology and Environmental Science Umeå University Linnaeus väg 6 90187 Umeå Sweden

[5] Department Lake Research, Helmholtz Centre for Environmental Research – UFZ, Brückstrasse 3a, 39114 Magdeburg, Germany

[6] Group of Molecular Microbial Ecology, Institute of Aquatic Ecology, University of Girona, Campus de Montilivi, 17071 Girona, Spain.

[7] Limnology, Department of Ecology and Genetics, Evolutionary Biology Centre, Uppsala University, Norbyvägen 18D, 75236 Uppsala, Sweden.

*Corresponding authors: Biel Obrador (obrador@ub.edu), Núria Catalán (ncatalangarcia@gmail.com)

Contents:

1. **Hydrological year and hydroperiod of the ponds**
2. **Characterization of organic matter**
3. **PLS analysis variables and VIP values**
4. **Microbial community composition**
5. **Supplementary Methods: Sequencing and phylogenetic analysis**

S1. Hydrological year and hydroperiod of the ponds

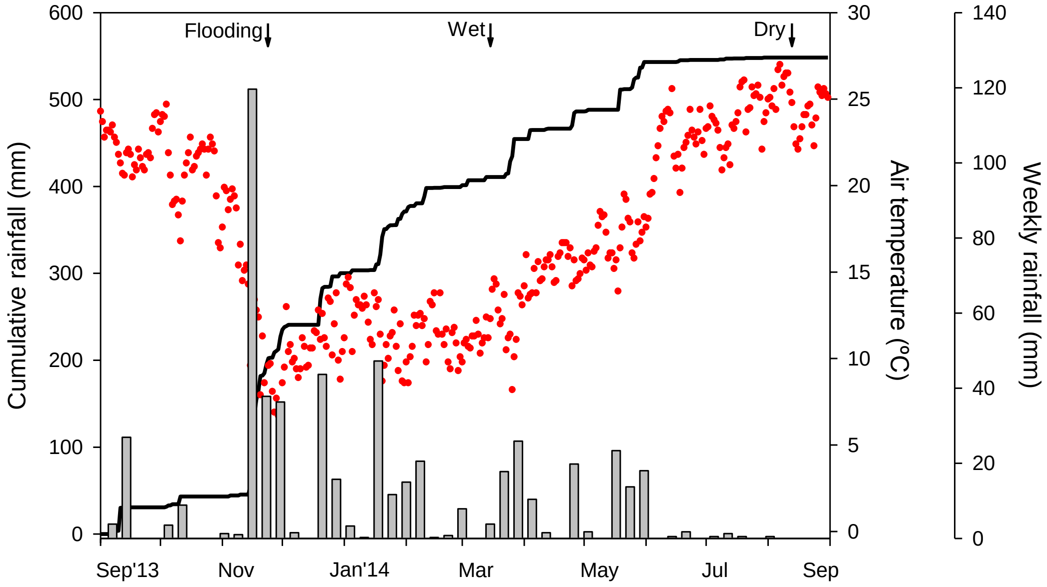

Fig. S1. Seasonal evolution of cumulative precipitation (bars), weekly rainfall (black line), and air temperature (red dots) during the hydrological year of 2014 (from September 2013 to September 2014) in the island of Menorca. The arrows indicate the samplings corresponding to the flooding, wet and dry phases of the ponds.

Table S1. Percent surface area of each of the three habitats for each pond across the sampled periods. *Denotes systems classified as ephemeral based on their hydroperiod (Ref. 46)

	Flooding phase			Wet phase			Dry phase		
		Dry habitats			Dry habitats			Dry habitats	
Pond	Inundated habitat (%)	Emerged bare sediments (%)	Emerged vegetated sediments (%)	Inundated habitat (%)	Emerged bare sediments (%)	Emerged vegetated sediments (%)	Inundated habitat (%)	Emerged bare sediments (%)	Emerged vegetated sediments (%)
Son Morell*	15	20	65	9	32	59	0	10	90
Curniola	0	71	29	100	0	0	0	71	29
Verda d'Algaiarens	24	0	76	58	21	21	0	5	95
Torrellafuda	13	24	63	21	0	79	0	0	100
Mal Lloc	39	0	61	100	0	0	0	5	95
Verda des compte*	0	100	0	0	60	40	0	100	0
Armaris	0	0	100	90	0	10	0	0	100
Sa Mesquida	78	11	11	96	0	4	0	0	100
Cap Negre*	28	0	72	1	84	15	0	0	100
Marina Curniola*	0	55	45	20	24	56	0	55	45

S2. Characterization of organic matter

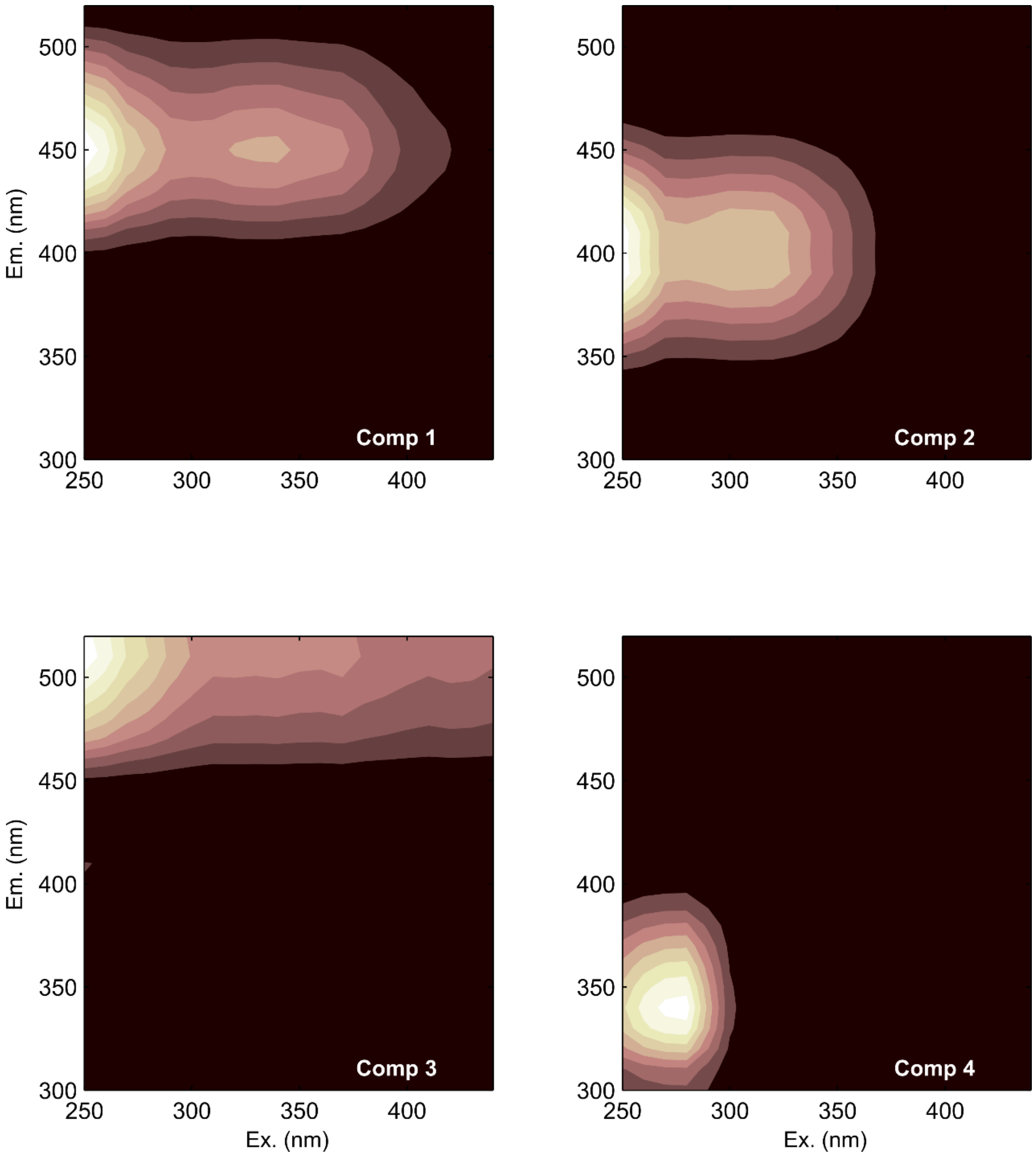

Fig. S2. Fingerprint of the four components validated in the PARAFAC model, referred as C1-C4 in the main text. A description is provided in Table S2.

Table S2. Components identified in the PARAFAC model and results of the query on the OpenFluor database (http://www.openfluor.org, January 2016). The matched components and corresponding model (TCC = 96%) are shown. The description of the models in our model is based on those previous studies.

Components	N*	Components and PARAFAC models* matched	Description/ Classification
**C1**	2	C1 (FCE)C1 (Model A)	Ubiquitous humic-like, terrestrially derived organic matter, similar to peaks A+C
**C2**	13	C2 (DONKEY); C2 (JapanSea); C2 (Model A); C2 (Model C); C2 (Model D); C2 (Omdrev); C2 (Recycle_PC); C2 (Recycle_WRAMS); C3 (Recycle_RH); C3 (SAB6_2001_2005); C4 (Horsens5); C6 (Antarctic)	Humic-like, terrestrially delivered reprocessed organic matter, microbial humic-like fluorescence, similar to peak M.
**C3**	9	C1 (NeusePOMDOM); C2 (ONR); C2 (SharkBay); C2 (TropicalRivers); C2 (Umeå); C2 (drEEM); C2 (graeber2012); C3 (PARTNERS); C5 (FCE)	Terrestrial humic-like, ubiquitous fulvic acid, reduced terrestrial semi-quinone fluorophore (SQ1)c
**C4**	5	C1 (NeuseBEPOM); C2 (CorrOrganicCompounds2); C5 (GoMaine); C5 (NeusePOMDOM); C6 (Quebec Boreal)	Protein-like, microbial derived or freshly produced organic material

*N denotes the number of models listed below matched with our PARAFAC model.

Antarctic	Stedmon C.A. et al. 2011. J. Geophys. Res. 116(G03027)1-9
CorrOrganicCompounds2	Wünsch UJ et al. 2016. Front. Mar. Sci. 3:9
DONKEY	Stedmon, C. A et al. 2007. Mar. Chem. 104: 227-240.
drEEM	Murphy, K.R. et al. 2013. Anal. Methods, 5: 6557-6566
FCE:	Yamashita, Y. et al. 2010. Ecosystems 13: 1006-1019.
GoMaine	Cawley et al., 2012. Mar. Pollution Bulletin, v64 : 1678-1687
graeber2012	Graeber, D. et al. 2012. Sci. Tot. Environ.,438 : 435-446
Horsens5	Murphy, K.R. et al. 2014, in: Coble, P. et al. (Eds.). Aquatic organic matter fluorescence. Cambridge University Press, New York.
JapanSea	Tanaka, K. et al. 2014. Sci. Rep. 4, 5292.
Quebec Boreal	Lapierre J. F. and del Giorgio P. A., 2014. Biogeosciences, 11: 5969–5985
Model A , C and D	Shutova et al., 2014. Water Res. 54: 159-169
NeuseBEPOM	Brym, A. et al. 2014. Mar. Chem., 162 : 96-113.
NeusePOMDOM	Osburn, C.L. et al. 2012. Environ. Sci. Technol. 46: 8628−8636
Omdrev	Kothawala, D. N. et al. 2013. Glob Chang Biol.20: 1101-1114
ONR	Osburn C.L. et al. 2011. Mar. Chem., 126: 281-294
PARTNERS	Walker S. A. et al. 2014. J. Geophys. Res. Biogeosci., 118: 1689-1702.
Recycle_PC, RH and WRAMS	Murphy, K.R. et al. 2011. Environ Sci Technol 45: 2909-2916
SAB6_2001_2005	Kowalczuk, P et al. 2009. Mar. Chem. 113: 182-196
SharkBay	Cawley et al., 2012. Mar. Freshwater Res. 63: 1098-1107.
TropicalRivers	Yamashita, Y. et al. 2010. J. Geophs. Res. 115, G00F10.
Umeå	Stedmon, C.A. et al. 2007. Environ. Sci. Technol. 41: 7273-7279.

S3. Variables in the PLS analysis and VIP values

Table S3.1. Drivers of the total CO_2 flux (T-FCO_2) determined by the (PLS) analysis. Variables are ordered by decreasing VIP (variable importance to the projection, shown with their ± SE). The analysis explained 92% of the variance in the T-FCO_2. Only the highly and moderately influential variables (i.e. VIP > 0.8) are shown.

**Abbreviation**	**Variable**	**VIP**
SI_C2	Inundated habitat, component 2 (%)	1.74 ± 0.71
SI_%MO	Inundated habitat, organic matter content (LOI; %)	1.58 ± 0.19
W_TN	Water, total nitrogen (mgN L^{-1})	1.28 ± 0.69
SE_%MO	Emerged sediment, organic matter content (LOI; %)	1.24 ± 0.42
SE_ T^a	Emerged sediment, temperature (ºC)	1.23 ± 0.37
W_Alk	Water Alkalinity (meq L^{-1})	1.22 ± 0.98
SE_DOC_Extract	Emerged sediment, DOC extract (WEOM; mg L^{-1})	1.21 ± 0.46
SI_C4	Inundated habitat, component 4 (%)	1.19 ± 0.54
SI_C1	Inundated habitat, component 1 (%)	1.19 ± 0.52
SE_ RH	Emerged sediment, relative humidity (%)	1.17 ± 0.92
W_DOC	Water, DOC (mg L^{-1})	1.11 ± 0.64
Env_Depth	Depth of the pond (cm)	1.03 ± 0.61
SE_HIX	Emerged sediment, humification index	1.01 ± 0.24
SE_C1	Emerged sediment, component 1 (%)	0.89 ± 0.24
W_BIX	Water, biological index	0.85 ± 0.57
SI_C3	Inundated habitat, component 3 (%)	0.84 ± 0.54
W_Chla	Water, Chl-a (µg L^{-1})	0.82 ± 0.57
SE_C4	Emerged sediment, component 4 (%)	0.82 ± 0.29

Table S3.2. Drivers of the CO_2 flux from inundated habitats (W-FCO_2) determined by the (PLS) analysis. Variables are ordered by decreasing VIP (variable importance to the projection, shown with their ± SE). The analysis explained 86% of the variance in the W-FCO_2. Only the highly and moderately influential variables (i.e. VIP > 0.8) are shown.

**Abbreviation**	**Variable**	**VIP ± SE**
HIX	Humification index	1.45 ± 0.14
C4	Fluorescence component 4 (%)	1.35 ±0.14
C1	Fluorescence component 1 (%)	1.35 ± 0.14
S_R	Absorbance slope ratio	1.24 ± 0.22
Cond.	Conductivity	1.21 ± 0.40
DOC	Dissolved organic carbon (mg L^{-1})	1.06 ± 0.31
C2	Fluorescence component 2 (%)	1.06 ± 0.34
C3	Fluorescence component 3 (%)	1.05 ± 0.15
BIX	Biological Index	1.05 ± 0.12
O_2	Oxygen concentration (mg L^{-1})	1.00 ± 0.32
TFDOM	Total fluorescence (Raman units)	0.90 ± 0.13

S4. Microbial community composition

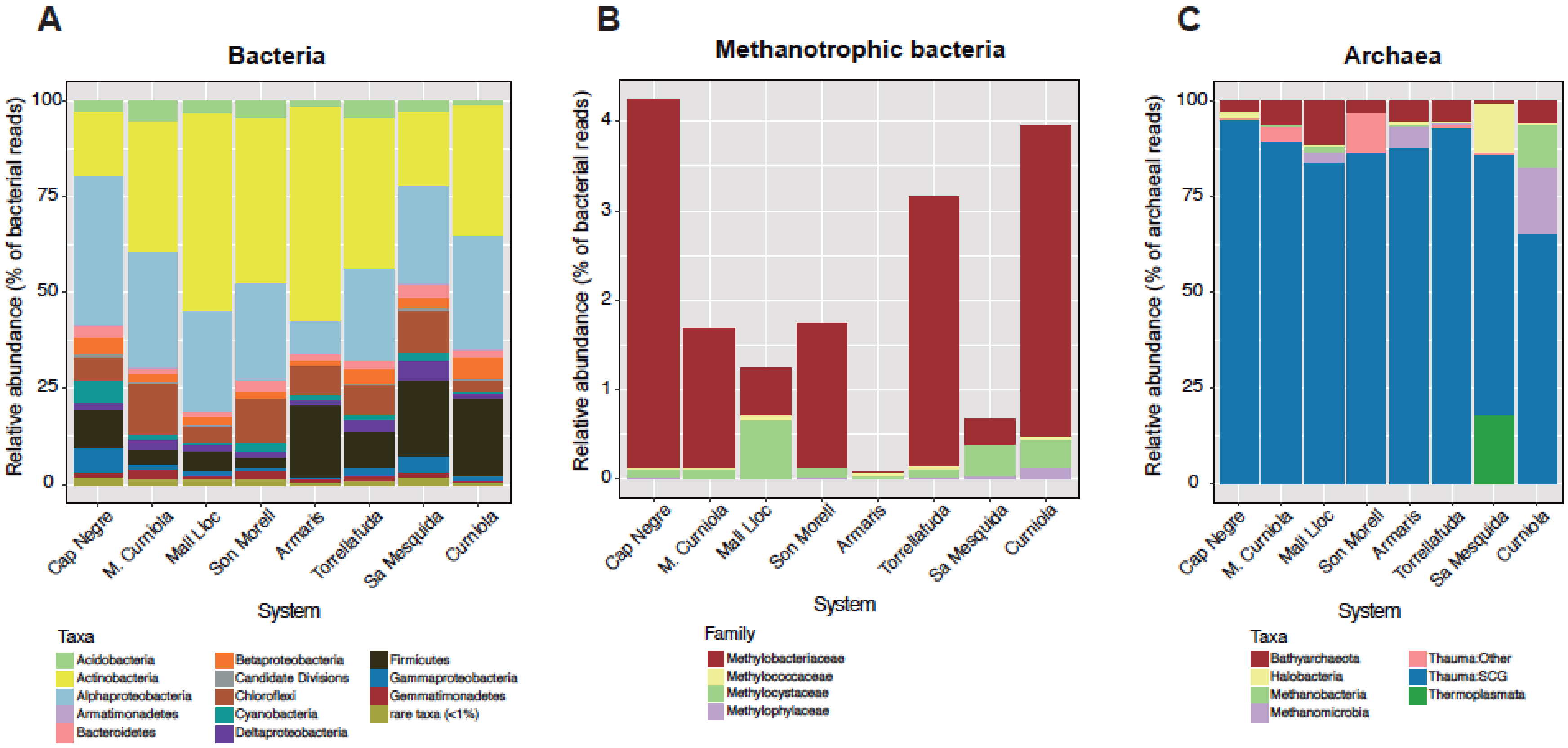

Fig. S4. Composition of a) bacterial, b) methanotrophic bacterial and c) archaeal sediment communities in the studied ponds.

Table S4. Distribution of (A) bacterial and (B) archaeal sequence reads across studied systems. The number of observed OTUs and estimators of richness (Chao1) and diversity (Shannon) are also shown. The number of sequences per sample was normalized to 28,000 and 12,000 for Bacteria and Archaea, respectively, to avoid biases due to different sequencing effort across samples.

A) Bacteria

Total reads: 512,136
Total OTUs: 8,621

System	Number of reads	Observed OTUs	Chao1[a]	Shannon[b]	Phylogenetic Diversity[c]
Armaris	29,142	1,296	1623	7.9	61.1
Cap Negre	121,157	2,442	3562	9.6	107
Curniola	28,910	1,443	1843	8.7	63.4
Mal Lloc	32,619	1,565	1860	9.1	70.4
Marina Curniola	80,380	2,139	2985	9.0	93.2
Sa Mesquida	151,517	1,527	1763	9.3	87.5
Son Morell	31,954	1,771	2352	8.3	79.3
Torrellafuda	36,457	1,635	1945	9.2	77.2

B) Archaea

Total reads: 447,892
Total OTUs: 240

System	Number of reads	Observed OTUs	Chao1[a]	Shannon[b]	Phylogenetic Diversity[c]
Armaris	35,713	104	112	4.8	3.9
Cap Negre	24,967	80	88	3.3	2.9
Curniola	63,184	109	113	5.3	3.8
Mal Lloc	83,789	100	112	3.9	4.1
Marina Curniola	12,494	73	82	4.4	2.5
Sa Mesquida	51,189	140	154	4.1	8.5
Son Morell	83,937	69	71	4.0	1.2
Torrellafuda	92,619	83	94	3.8	2.6

[a]Chao, A. 1984. *Scandinavian J. Stat.* 11:265-270)
[b]Shannon, C. E. 1948. The Bell Syst.Technic. J., 27: 379–423 and 623–656.
[c]Faith, D.P. 1992. *Biol. Conserv.* 61:1–10

S5. Supplementary Methods: *Sequencing and phylogenetic analysis*

Genomic DNA from sedimentary communities was used as a template in PCR reactions using primers 28F (Sundquist et al., 2007) and 388R (Turnbaugh et al., 2009) for bacterial 16S rRNA genes and primers 517F (Baker et al., 2003) and 909R (*a.k.a.* 890aR, Burgraff et al., 1997) and then analyzed through MiSeq PE2x250 Illumina chemistry at the RTLGenomics (Lubbock, TX, USA). Both primer pairs were complemented with Illumina-adapters and sample-specific barcodes. Raw sequence dataset was pre-processed at RTLGenomics facilities to reduce noise and sequencing artefacts as well as to merge forward and reverse pairs. Merged sequences were quality-filtered excluding sequences with quality scores <25, with lengths <250 bp or >400 bp, and with erroneous barcodes, ambiguous bases and homopolymers in QIIME (Caporaso et al. 2010a). De-novo and reference-based chimera checking, clustering into Operational Taxonomic Units (OTU, 97% cutoff), identification of representative OTU sequences and construction of OTU table were also carried out using default parameters in QIIME. OTUs with less than 4 members were removed from further analyses. Representative sequences from each OTU were then aligned to the Greengenes imputed core reference alignment (DeSantis et al., 2006) using PyNAST (Caporaso et al., 2010b). Taxonomical assignments for each OTU were carried out in QIIME using the BLAST method and the QIIME-formatted version of the SILVA 111 reference database. For community analysis, the number of sequences in each sample was normalized by randomly selecting a subset of 3,000 sequences from each sample to standardize sequencing effort across samples and to minimize any bias due to different number of total sequences. QIIME was also used to calculate α-diversity indicators of richness (Observed richness and Chao1) and diversity (Shannon index and Phylogenetic Diversity, Faith (1992)) for bacterial and archaeal communities and to calculate community similarity among sites (β-diversity) using unweighted and weighted UniFrac distances (Lozupone and Knight, 2005). All raw bacterial and archaeal DNA sequences retrieved in this study are publicly available through the Sequence Read Archive (SRA) database under accession SRP126916 (https://www.ncbi.nlm.nih.gov/sra).

References

Baker, G. C. et al. *J. Microbiol. Methods* 55: 541-555 (2003)

Burggraf S. et al. *Internat. J. Syst. Bacteriol*. 47: 657-660 (1997)

Caporaso J. G. et al. *Nat. Methods*, 7: 335–336 (2010a)

Caporaso J. G. et al. *Bioinformatics*, 26: 266–267. (2010b)

DeSantis T. Z. et al. *Appl. Environ. Microbiol.*, 72: 5069–5072 (2006).

Faith D. P. *Biol. Conserv*., 61: 1–10 (1992)

Lozupone C. and Knight R. *Appl. Environ. Microbiol.*, 71: 8228–8235 (2005).

Sundquist A. et al. *BMC Microbiology* 7: 108 (2007)

Turnbaugh P. J. et al. *Nature* 457: 480–484. (2009)
